# Emerging trends in the cystatin C sensing technologies: towards better chronic kidney disease management

**DOI:** 10.1039/d4ra07197b

**Published:** 2025-02-14

**Authors:** Jeethu Raveendran, Dhanya Gangadharan, Jagadeesh Bayry, P. Abdul Rasheed

**Affiliations:** a Department of Biological Sciences and Engineering, Indian Institute of Technology Palakkad Palakkad Kerala 678623 India abdulrasheed@iitpkd.ac.in; b Department of Biotechnology, Sahrdaya College of Engineering and Technology Thrissur 684002 Kerala India; c Department of Chemistry, Indian Institute of Technology Palakkad Palakkad Kerala 678623 India

## Abstract

Cystatin C (CysC), a protein, has replaced creatinine as a biomarker of kidney function and other diseases and has led to a surge in the research on the development of efficient CysC biosensors. The current CysC sensing technologies are remarkable in terms of selectivity and reproducibility. However, the complexity, cost, and space requirements of these methods render them unsuitable for real-time monitoring or point-of-care (PoC) implementations in healthcare settings. This review discusses the most recent developments in the field of CysC biosensing and to the best of our knowledge, this is the first focused review exclusively on CysC biosensing modalities. Our goal is to provide a thorough overview of the current state of CysC biosensors, and presenting mechanisms related to biosensor recognition and transduction. The review starts with clinical significance of CysC detection followed by detailed analysis of different CysC biosensing methods with emphasis on the necessity of PoC monitoring of CysC. We have also highlighted current challenges and an outlook on future perspectives. We anticipate that this study will play a key role in the understanding the working principle of CysC sensors and will aid in the designing of new efficient sensing modalities for the detection of CysC.

## Abstract 2

This review focuses clinical significance of CysC detection followed by detailed analysis of different CysC biosensing methods with emphasis on the necessity of PoC monitoring of CysC.

## Graphical abstract

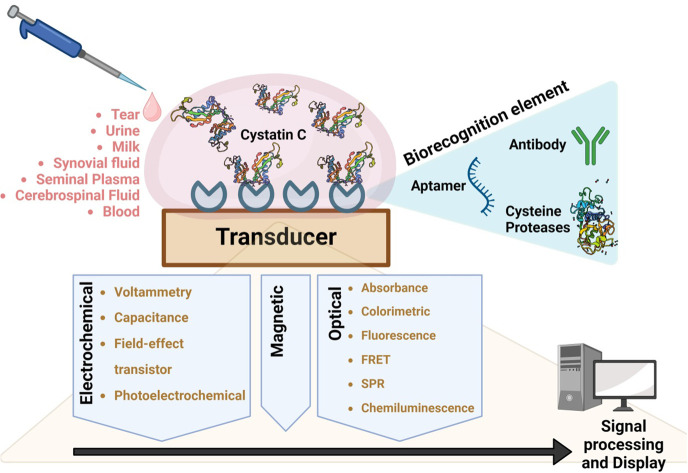

## 1. Introduction

Millions of individuals worldwide suffer from chronic kidney disease (CKD), and its incidence is increasing, particularly among elderly people.^1^ Renal impairment in the elderly has been linked to an increased risk of consequences such as heart failure, acute myocardial infarction and malfunctioning of other organs.^2,3^ The glomerular filtration rate (GFR), which is based on the blood levels of endogenous filtration indicators (mainly creatinine), is used for the clinical assessment, identification, evaluation, and management of CKD.^4^ As the creatinine concentrations significantly vary with the physiological conditions of the patients, creatinine-based estimation of GFR should be used only for patients with stable creatinine concentrations. Due to its insensitivity in identifying mild to moderate changes in GFR, the accurate interpretation of serum creatinine in a clinical setting presents different challenges and considerable research is ongoing for a more accurate biomarker of CKD. Towards this aspect, it is established that the possible alternate biomarker for creatinine is cystatin C (CysC), which shows promise as a convenient measure of GFR.^5^

Human CysC is a stable basic 13 kDa protein composed of a single polypeptide chain with 120 amino acid residues and two disulfide bonds. CysC consist of a short alpha helix and a long alpha helix, which lies across five-stranded beta sheet. About 120 residues and two disulfide bridges define Family II, which includes cystatin C and S, and their derivatives. It is a critical cysteine protease inhibitor routinely generated by nearly every nucleated cell in the body and the production of CysC is irrespective of one's gender, age, or muscle mass. Because of its low molecular weight, it can easily cross the glomerular membrane and undertake near-complete metabolism within the tubular cells at the proximal end of the kidneys.^6^ The level of CysC in the blood will be in the normal range when kidneys functions well and the level of CysC varies if kidneys are not functioning well. These distinct characteristics of CysC make them as the best biomarker for estimating the GFR, capable of detecting even modest changes in GFR which can be used to detect early kidney disease and aid in the diagnosis of CKD.^7^ In addition to estimating the GFR, the CysC levels have been shown to be a prognostic marker for a variety of illnesses, such as tumors, polycystic ovarian syndrome (PCOS), brain diseases, cardiovascular problems, and endocrine disorders.

## 2. Clinical relevance of CysC estimation from biosamples

CysC is found in all body fluids of human, including tear, urine, milk, synovial fluid, seminal plasma, and blood. In healthy people, the range of the blood CysC content is 0.8–1.2 mg L^−1^.^8^ The possibility of using CysC as a marker of kidney function is well studied and it has been established that CysC detection offers more sensitive and selective method for the early identification of renal diseases when compared to other markers such as creatinine and urea.^8–11^ Renal failure is a severe complication in patients with liver cirrhosis and its diagnosis based on serum creatinine cannot be used to measure of renal function in such patients. CysC has been used for the prognosis of renal dysfunction, hepatorenal syndrome, acute-on-chronic liver failure; and the monitoring of CysC may help in identification of the patients at risk and initiation of early clinical management.^12^

It has also been shown that fetal urine CysC correlates with postnatal kidney function at birth and serves as a biomarker of fetal renal tubular damage. The range of reported CysC levels in amniotic fluid is 0.06 to 2.68 mg L^−1^ in healthy full term pregnant women.^13^ The amniotic fluid samples from fetal uropathies and those with normal kidney functions display significant differences in the CysC values while creatinine levels were found to be very similar between these two groups.^14^ The CysC based diagnosis shows 96% diagnostic accuracy when it comes to distinguishing between fetuses with obstructive uropathies and those with normal kidney function, while creatinine has only 62% screening precision.^15^ Patients with hypertension and coronary heart disease show elevated CysC levels, which correlate with the severity of the illness.^16^ Recent research has also revealed that elevated CysC in the patients with acute heart failure and chronic kidney disease acts as an independent risk factor for cardiovascular death.^17^

The CysC levels in the urine are typically extremely small, between 0.03 to 0.30 mg L^−1^ and the urine CysC concentrations in healthy individuals do not significantly change with the age or muscle mass.^18^ An increase in the concentration of CysC in urine might be due to either a disturbed tubular reabsorption mechanism or a higher serum level over its reabsorptive threshold. Urinary CysC concentrations in such individuals can increase up to 200 times. The monitoring of CysC also plays a significant role in the prognosis of type 2 diabetic nephropathy as serum CysC gives more accurate values than serum creatinine.^19^ Additionally, the CysC levels are also linked with the thyroid glands functioning as mild thyroid dysfunction significantly alters CysC levels.^20^ CysC levels exhibit a more pronounced reduction in cases of hypothyroidism and it is higher in hyperthyroidism. Consequently, this implies that GFR is likely to be underestimated in hyperthyroidism and overestimated in hypothyroidism when considering CysC levels.^21^ Therefore, thyroid function parameters have to be considered when CysC is used to evaluate the kidney function.

It has long been known that the CysC is the main cysteine protease inhibitor in the cerebral spinal fluid (CSF). The CysC levels in the CSF of healthy adults are roughly 5.8 mg L^−1^, which is 5.6 fold greater than in plasma.^22^ Patients with disorders like meningitis, encephalitis, chorioretinitis, and myelitis have been found to have higher concentrations of CysC in their CSF, whereas patients with multiple sclerosis, acute Guillain–Barré syndrome, and chronic inflammatory demyelinating polyneuropathy had significantly lower levels in their CSF.^23^ This drop in concentration could be due to decreased CysC synthesis or an increased degradation in inflammatory lesions of the central nervous system. Another study evaluated the possibility of CysC as a CSF biomarker for pain in humans based on the hypothesis that increased expression of CysC in the dorsal horn leads to its increased secretion in CSF. They confirmed that pain induces the production and discharge of CysC in the dorsal spinal cord and it overflows into the CSF so that the level will be high during active pain states.^24^

Proteases and their inhibitors are in equilibrium, and this balance governs most normal and pathological processes.^25^ It was discovered that CysC might be involved in regulating the expression of cathepsin B, a cysteine protease primarily present in fast spreading cancer cells.^26^ The less common cathepsin B/CysC complexes in the sera of cancer patients compared to healthy controls or patients with benign diseases suggested an imbalance between the enzyme and its inhibitor in these patients.^27^

**Fig. 1:**
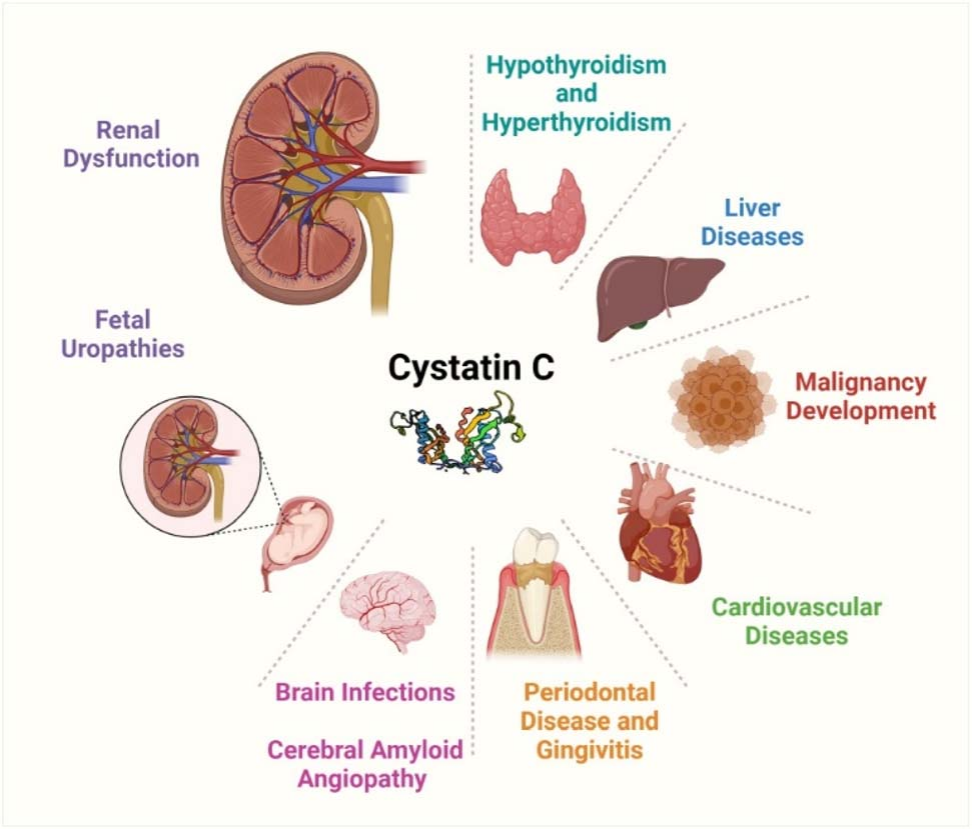
Schematics showing the clinical significance of CysC in various disorders. The authors have drawn the figure using https://www.BioRender.com.

It was discovered that seminal plasma had a mean CysC concentration of 51.0 mg L^−1^. This is approximately 36 times greater than that found in healthy adult blood plasma and is due to the larger synthesis of CysC by the male genital tract glands.^28^ Similarly, the CysC level is upregulated in inflammatory conditions, such as periodontal disease and gingivitis.^29,30^ Compared to periodontally healthy individuals, those with gingivitis or periodontitis show approximately 1.3 times greater amounts of CysC in the saliva.^31^ In cerebral amyloid angiopathy, a disease characterized by amyloid beta-peptide deposits within blood vessels of brain, hemorrhage is highly predicted by the degree of CysC accumulation.^32^ Also, increased serum levels of CysC are associated with possible future stroke events in the elder patients especially with carotid atherosclerosis.^33^ Similarly, the CysC level is higher for amyotrophic lateral sclerosis, which is a fatal neurologic disease characterized by progressive motor neuron degeneration.^34^ The higher value of CysC in comparison with healthy controls highlights the severity of the disease in terms of faster progression rate and shorter survival. The overall clinical significance of CysC is summarized in Fig. 1.

Based on these reports, it is clear that CysC concentrations in body fluid are strongly correlated with a variety of pathologies. The majority of the reports highlight the importance of CysC for monitoring renal impairment along with other mentioned diseases. A number of techniques including conventional methods and newly introduced methods are used in various clinical settings to monitor CysC in various body fluids. The following section discusses these conventional and newly introduced methods for the sensing of CysC.

## 3. Conventional sensing methods for the detection of cystatin C

Conventional methods for CysC detection majorly rely on particle-enhanced nephelometric immunoassay (PENIA), particle-enhanced turbidimetric immunoassay (PETIA), and Enzyme-Linked Immunosorbent assay (ELISA). All these techniques use immunochemistry as principle by antigen–antibody complex formation. The basis of PENIA is the development of an antigen–antibody complex, which causes light scattering.^35^ This method involves incubating biologic specimens containing CysC with microspheres coated with anti-CysC antibodies and the light scattering is proportional to the concentration of CysC. The similar idea underlies in PETIA, which quantifies variations in absorbance following immunocomplex formation.^36^ The ELISA technique employs two antibodies: a biotinylated polyclonal antibody to CysC and a monoclonal antibody, and this sandwich structure of the test seems to increase the specificity of this method.^37^

PETIA and PENIA methods have been widely explored and studies show that PENIA methods for the detection of CysC are more sensitive.^38^ PENIA methods have been reported to exhibit optimum performance in dilute solutions, thus making it suitable for pediatric population where small volumes of the samples are preferred. The detection of CysC in the same blood samples with the PETIA assay and the PENIA method have reported differences in values,^35,39^ which could be attributed to difference in reactivity to CysC antibodies, or difference in standards or substrates.^39^ Most of the PETIA and PENIA studies use rabbit anti-human antibodies for the analysis. Jonkisz *et al.* compared the CysC values obtained by PETIA and PENIA assays in the dog by using 40 dogs in four groups with different stages of chronic kidney disease based on serum creatinine values.^40^ The serum CysC was measured using PETIA and PENIA, and the correlations with the serum urea and creatinine values were studied. The results confirmed the higher sensitivity and correlation with other accepted indicators of renal function in the values obtained in PENIA when compared to the PETIA. Another study was conducted to evaluate the performance of the analytical technique using two reagents for the detection of traceable urinary CysC by Bargnoux *et al.*^38^ Siemens reagents were used for PENIA and DiaSys reagents were used for PETIA for assessing their clinical relevance in healthy and individuals with tubular dysfunction. They found that results were comparable in both methods.

ELISA is a widely accepted method for measuring CysC levels due to its sensitivity and specificity.^41^ ELISA has been used as a method of measurement to prove the effect of non-renal factors like corticosteroids, which could lead to an increase in the plasma CysC levels without any effects on kidney function. The hypothesis was tested in rats by studying the effect of corticosteroids on the levels of CysC in their tissues and its effect on GFR.^42^ The data showed that corticosteroids may increase CysC levels in the plasma by promoting its production, without any decrease in GFR. Wilson *et al.* conducted quantitative ELISA to assess the plasma CysC levels in 104 patients diagnosed with ALS.^34^ They found that the CysC levels in amyotrophic lateral sclerosis patients were significantly elevated in plasma and diminished in CSF compared to healthy controls. Additionally, the CysC level in CSF is correlated to the rate of ALS disease progression, which suggests it can be used as a surrogate marker of disease progression and survival.

It is difficult to get efficient paired monoclonal antibodies against small molecules such as CysC, which can be used in clinical set ups and this led to use of hybridoma and phage display techniques to obtain monoclonal antibodies against CysC. Jiang *et al.* introduced efficient paired monoclonal antibodies against CysC by establishing a new double-antibody-sandwich ELISA (DAS-ELISA)-based measurements.^43^ Mommaerts *et al.* developed a double indirect sandwich ELISA to estimate the total CysC present in the biological fluids (both cleaved and uncleaved forms).^44^ The ratio of cleaved and uncleaved concentrations was used to evaluate the extent of CysC cleavage using this novel method.

Despite being sensitive and rapid tests, automated PETIA, PENIA and ELISA are limited in their application in ordinary clinical practice due to costly procedures and use of specific immunoassay apparatus. The requirement for relatively expensive test kits and clunky plate readers, on the other hand, restrict their applicability for point-of-care (PoC) diagnostics. To overcome these constraints, PoC biosensors based on new biosensing methods can be utilized for CysC monitoring.

## 4. CysC sensing based on bio recognition methods

Based on the literature survey regarding the developed CysC sensors, almost all of the sensors utilized the specific binding of antibodies, aptamers, or cysteine proteases. Researchers were able to develop sensors with a variety of transduction pathways by using their selective recognition capabilities. The following sections address the research on how the specific antibodies or aptamers or cysteine proteases demonstrate their applicability as bio-recognition elements. There are mainly three bio-recognition methods have been used for the CysC sensing such as antibodies, aptamer and cysteine proteases based recognitions.

### 4.1. Antibodies as bio-receptor

Numerous studies have focused on the development and characterization of monoclonal antibodies specific to CysC.^45–47^ The interactions between CysC and their specific monoclonal antibodies have been clearly studied by different research groups. The anti-CysC antibodies have been extensively used for the detection of CysC by various techniques such as immunohistochemistry, western blot, immunocytochemistry, Luminex, and ELISA. Among these techniques, antibodies have long been employed in the identification of CysC through conventional ELISA kits.^41,43,48^ In addition, the interactions between monoclonal antibody and CysC were studied in detail to identify the CysC epitopes.^49–51^ The antibody-based detection of CysC have been done by both electrochemical and optical sensing, and these techniques are discussed in the following sections.

### 4.2. Aptamer based recognition

The short synthetic single-stranded DNA or RNA molecules known as oligonucleotide aptamers are highly useful in clinical diagnostics, and therapeutic applications because of their broad molecular binding capacity. Lack of immunogenicity, small size, and high specificity of aptamers make them viable substitutes to antibodies.^52^ Aptamer-based technologies are extremely important in biomarker discovery in which the specific binding of aptamer and biomarker is used for the detection. The two important approaches for identifying the biomarker are cell-systematic evolution of ligands by exponential enrichment (Cell-SELEX),^53^ which is mainly utilized to identify biomarkers on the cell membrane surface, and SOMAScan technology,^54^ an objective biomarker detection technique, which can analyze multiple proteins in complex biological samples. In general, aptamers are identified through normal SELEX, which is an iterative selection–amplification process. It is typically carried out using purified target molecules, and whole live cells can be the selection targets in this case. However, in Cell-SELEX, and cell-specific aptamers can be obtained the targets are cell surface transmembrane proteins even though these are difficult to purify.

A competitive Enzyme-Linked Aptamer Sorbent Assay (ELASA) employing single-stranded DNA aptamers was developed to measure the levels of CysC in serum samples.^55^ The chosen aptamer exhibited a low dissociation constant (*K*_d_) of 65.5 ± 0.007 nM and limit of detection (LOD) of the assay was found to be 216.077 pg mL^−1^. The analytical outcomes of this method were in line with those from widely used commercial kits for the CysC detection. In another work, Lopez-Silva *et al.* assessed the use of SOMAscan technology, which is an aptamer-based technology, for the detection of CysC from kidney disease patients using immunoassays as the gold standard.^56^ The study suggested that the SOMA scan is an efficient and relatively reliable technique for quantifying CysC and to correlate clinical outcomes. Recently, Dubin *et al.* also studied the analytical and biological variability of the SomaScan V4 aptamer proteomic assay in plasma samples from patients with moderate to severe CKD.^57^ The assay's robust performance was demonstrated by low intra-assay and within-subject variability, as well as high Rho correlations between aptamer and traditional CysC assays. These correlations were comparable to those reported in individuals without CKD.

### 4.3. Cysteine proteases based recognition

It was established that CysC is a critical cysteine protease inhibitor and it carries out peptide bond hydrolysis using histidine and its reactive site cysteine as the catalytic nucleophile.^58^ The name cystatin has a linguistic association with its inhibitory action. Cystatin's “stat” is derived from the Latin word “sisto,” which means “to arrest,” highlighting the ability of cystatins to stop the action of cysteine proteases. The three families that make up the cystatin superfamily are included in the comprehensive categorization of cysteine protease inhibitors, which was first described by Barrett *et al.* in 1986.^59^

Papain is a cysteine protease, which consists of a polypeptide chain with three disulfide bridges and a sulfhydryl group which are necessary for activity of the enzyme.^60^ A major group of the cysteine proteases are structurally related to papain and are therefore named as papain-like cysteine proteases. The proteins, which block papain-like cysteine proteases have been recognized in 1946 by D. Grob and CysC is one among them.^61^ When papain mixes with an excess of human CysC or chicken cystatin, an enzyme–inhibitor complex was formed immediately.^62^ Abrahamson *et al.* investigated the formation of inhibitor complex and identified that the peptidyl bond of the conserved glycine residue in human CysC and chicken cystatin might be the substrate-like inhibitory reactive site.^62^ They also proposed that the inhibition of cysteine proteinases might be due to the simultaneous interactions with the inhibitory reactive sites of the protease and cystatins. Lindahl *et al.* studied the interaction between recombinant human CysC and the cysteine proteinases papain by spectroscopic, kinetic and equilibrium methods.^63^ The dissociation equilibrium constant for binding CysC to papain was approx. 11 fM. The affinity between papain and CysC decreased with increasing size of the inactivating group, which is similar to earlier observations with the chicken cystatins. The CysC–papain interactions were extensively used for the designing of biosensors for the detection of CysC in conjunction with most of the new biosensing based transduction mechanisms which are discussed in the following sections.

## 5. New biosensing methods for the detection of cystatin C

This section discusses the recent advances in the CysC biosensing based on transduction and biorecognition elements. Based on the transduction mechanism, the CysC sensors can be classified into electrochemical, optical and nuclear magnetic resonance (NMR) based sensors. All these methods use either antibody or cysteine proteases as the binding agent to the CysC.

### 5.1. Electrochemical methods for the detection of CysC

Electrochemical CysC biosensors mainly use different bio-recognition elements such as labeled or unlabeled antibodies and molecularly imprinted polymers to bind with CysC. The binding between CysC and antibodies are monitored using appropriate labeling mechanisms. The reported works on electrochemical CysC biosensors with these bio recognitions elements are discussed below.

#### 5.1.1. Immunoassays

**Fig. 2:**
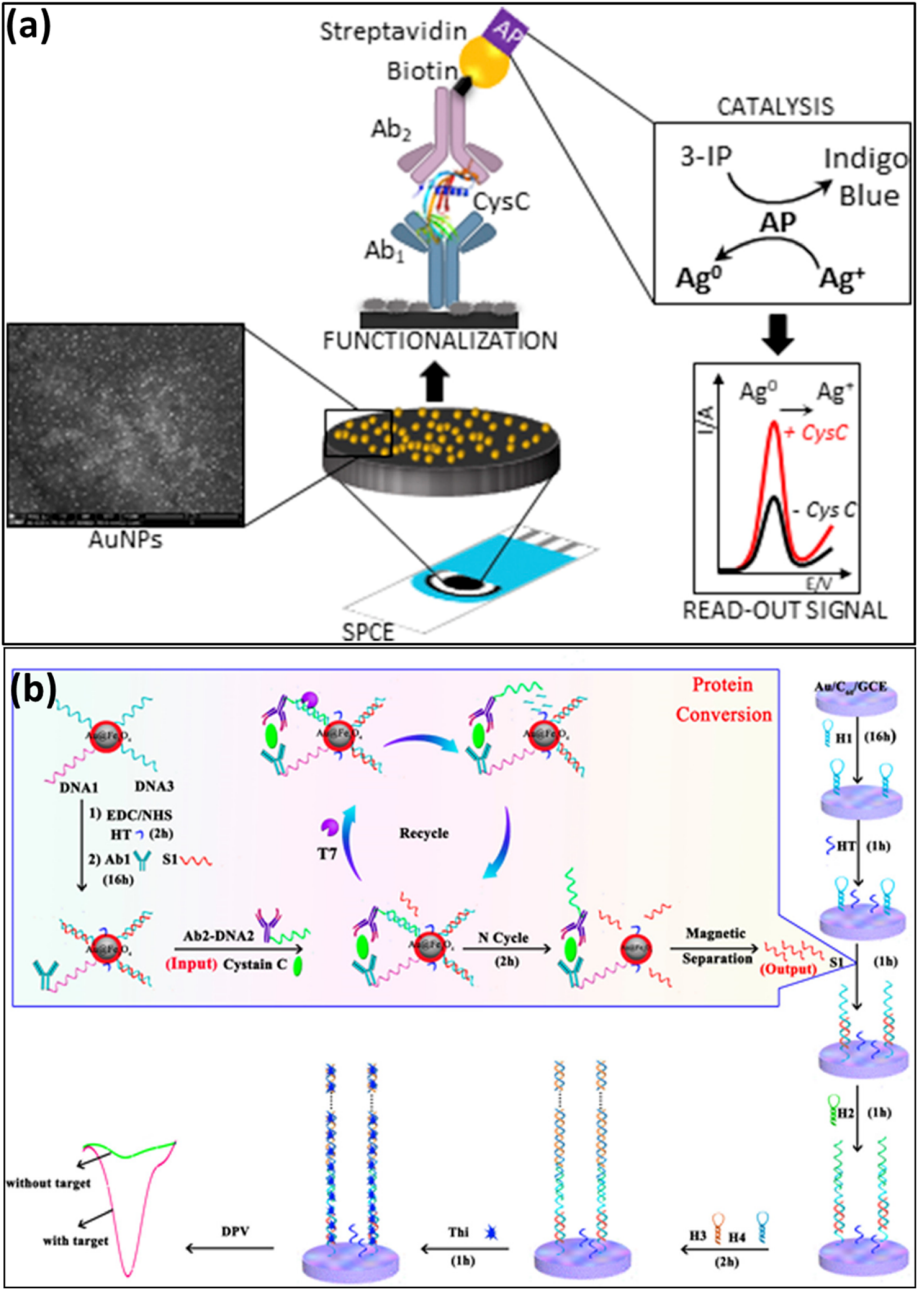
(a). Schematic representation of the sandwich-immunoassay with labeled enzyme alkaline phosphatase (AP) for catalyzing silver deposition reaction for the CysC detection. Reprinted with permission from ref. 64. Copyright © 2019 Elsevier B.V. (b) Electrochemical detection of CysC by protein converting strategy in combination with T7 Exo-assisted protein cyclic enzymatic amplification. Reprinted with permission from ref. 69. Copyright © 2016, American Chemical Society.

Both label free and label based immunoassays were used for electrochemical detection of CysC, which used specific antibody to CysC for the immobilization. A non-competitive sandwich-immunoassay with labeled enzyme alkaline phosphatase (AP) for catalyzing silver deposition reaction for the CysC detection was introduced by Lopes *et al.*^64^ Initially, the anti-CysC capture antibody was immobilized on gold nanoparticles (AuNPs)-modified screen printed carbon electrodes (SPCE). The biotin functionalized secondary anti-CysC capture antibody was added for the sandwich reaction in presence of CysC. This was followed by the addition of streptavidin modified enzyme alkaline phosphatase. A chemical reaction occurs when silver nitrate and 3-indoxyl phosphate (3-IP) are added to the assay. When AP hydrolyzes 3-IP, an indoxyl intermediate is produced, which oxidizes to indigo blue and lowers the Ag^+^ in solution. The metallic Ag that finally formed on the electrode surface was identified using voltammetry and is used for the quantification of CysC. Serum samples from patients with CKD were used for the real sample analyses, and the outcomes agreed with those of the Siemens-PENIA commercial golden standard test. The linear operating range of the sensor was 10 to 100 ng mL^−1^, and its detection limit was 6.0 ng mL^−1^. The schematics and working principle of this sensor are given in Fig. 2(a).

Another label-free electrochemical immunobiosensor for the rapid detection CysC has been developed by Krishnan *et al.* Here, anti-CysC antibody was immobilized on a carbon working electrode that had been modified with a graphene oxide-chitosan nanocomposite.^65^ The CysC binding to the antibody increased the charge transfer resistance of the electrode surface and this was evaluated by using ferricyanide as a redox mediator. The sensor detected CysC ranging from 1 to 10 mg L^−1^ with a detection limit of 7.8 μg L^−1^. The sensor was able to produce the response in just 420 s, which was shorter than conventional ELISA and other reported electrochemical sensors. The sensor response was evaluated with patient samples and found that the sensor can be employed used to predict the risk of developing diabetic retinopathy. In addition, the prediction accuracy of the sensor was marginally greater than that of the data acquired using the conventional ELISA approach. Similarly, a label-free electrochemical immunosensor for CysC was introduced by using ferrocene functionalized-graphene platform on a gold electrode.^66^ Here, the ferrocene acts as the redox active indicator of immunoassay production on the electrode surface. The complete sensor platform was created by drop-casting polyethyleneimine film followed by the addition of CysC. The addition of CysC decreases the current due to the electron transfer hindering by these adsorbed molecule and this was used for the detection of CysC. The sensor platform exhibited a response with a range of 0.1 to 1000 ng mL^−1^ and a LOD of 0.03 ng mL^−1^.

In another work, Chakraborty *et al.* prepared a novel nanostructure composite of ZIF-8-Cu_1−*x*_Ni_*x*_(OH)_2_@Cu, which was made by combining Cu shell on zeolitic imidazolate frameworks (ZIF-8) and Cu_1−*x*_Ni_*x*_(OH)_2_.^67^ In order to develop the CysC sensor, the poly(3,4-ethylenedioxythiophene) polystyrene sulfonate (PEDOT:PSS) coated indium tin oxide (ITO) electrode had been modified using the synthesized ZIF-8-Cu_1−*x*_Ni_*x*_(OH)_2_@Cu nanocomposite. Streptococcal protein G (SPG) was then used to functionalize the electrode, enabling it to bind the Fc region of anti-cystatin C. A concentration increase of CysC from 0.1 ng mL^−1^ to 1000 ng mL^−1^ increased the sensor's differential pulse voltammetry (DPV) current response, and the LOD was determined to be 33 pg mL^−1^. The developed sensor's excellent sensor performance may be attributed to its high surface coverage, excellent redox properties, improved electroactive sites, and synergistic catalytic activities. The sensor's clinical viability was confirmed through the measurement of CysC derived from human serum.

It is well known that papain is a cysteine protease, which can bind to CysC and this interaction was used for the fabrication of an electrochemical sensor for CysC.^68^ A screen printed electrode (SPE) with carboxyl activated multiwalled carbon nanotube electrode was used to modify the papain through binding between amino group of papain and carboxyl group on electrode surface by 1-ethyl-3-(-3-dimethylaminopropyl) carbodiimide (EDC)-*N*-hydroxysuccinimide (NHS) linking. The CysC–papain interaction mediated transduction of electrons by the sensor electrode was used to get the quantitative detection of CysC by using potassium ferricyanide as an indicator. The selectivity of the sensor was evaluated by using other urine-based biomarkers and found that there is no significant sensor response, which confirms the superior selectivity. The LOD of the sensor was found to be 60 ng L^−1^.

In a different approach, Chai *et al.* developed a novel immunoassay method for the CysC detection by using immunoreaction-induced DNA strand displacement and T7 Exonuclease (T7 Exo)-assisted protein cyclic enzymatic amplification.^69^ They have used Au@Fe_3_O_4_ as magnetic separator, which was labeled by conjugated DNA (DNA1) and the DNA substrate of T7 Exo (DNA3). This was followed addition of output DNA, antibody 1 (Ab1) and hexanethiol and incubation for 16 h. This resulted in the formation of DNA3-output DNA complex and DNA–Ab1 complex on the surface of Au@Fe_3_O_4_. This bio complex was incubated with DNA2–Ab2 complex and T7 Exo and CysC. The schematics showing the complete steps of sensor fabrication are given in Fig. 2(b). In the presence of CysC, the sandwich immunoreaction between DNA1–Ab1 and DNA2–Ab2 induces the proximity hybridization between DNA2 and DNA3 and thus displace output DNA from the output DNA/DNA3 duplex and leads to the formation of DNA2/DNA3 duplex. The T7 Exo digest the DNA2/DNA3 duplex with the release of DNA2, which acts as competing DNA to displace output DNA further. This output DNA strand was captured using an electrode modified with hairpin DNA, and a hybridization chain reaction was initiated on the electrode surface. The quantification of output DNA, which is the indirect measurement of CysC was done by using thionine as electron mediator. The linear range of the developed sensor was significantly larger, ranging from 0.01 pg mL^−1^ to 30 ng mL^−1^, with a low detection limit of 3 fg mL^−1^.

#### 5.1.2. Capacitive electrochemical techniques

**Fig. 3:**
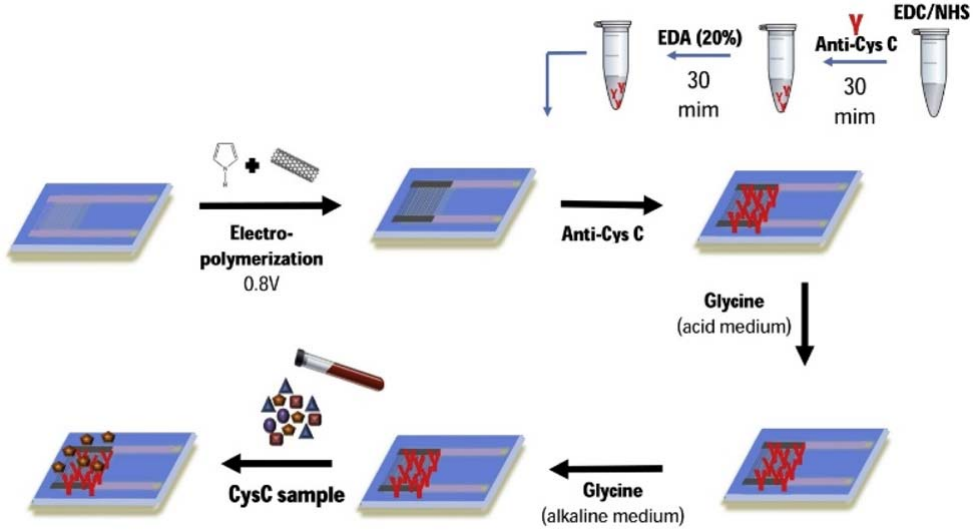
The schematics showing the stepwise modifications of the surface of the IDE immunosensor for the detection of CysC by measuring the double layer capacitance by the changes in phase angle. Reprinted with permission from.^70^ Copyright (2020), with permission from Elsevier.

An electrochemical immunosensor for CysC based on interdigitated electrodes (IDE) has been introduced by Ferreira *et al.*^70^ In this work, a polypyrrole/carbon nanotube nanohybrid sheet was incorporated on two gold electrodes of IDE to generate a supercapacitor. Covalent trapping with an ethylenediamine bifunctional agent and glycine blocking were used to immobilize CysC antibodies on the IDE. The schematic of the sensor fabrication is given in Fig. 3. The capacitive effect of antigen–antibody interaction was observed by double layer capacitance. The detection of CysC by this IDE immunosensor was monitored by evaluating the double layer capacitance by the changes in phase angle. The developed sensor was able to detect CysC in the range of 0–300 ng mL^−1^ with a LOD of 28 ng mL^−1^. The advantage of this work is the use of IDE for CysC sensing, which can be effectively used in the PoC testing due to its small size and better sensitivity in comparison with screen-printed electrochemical sensing.

#### 5.1.3. Molecular imprinted polymers

An artificial antibody was developed and employed for the detection of CysC by modifying a molecularly imprinted polymer (MIP) on a SPCE.^71^ The MIP was made by electro polymerizing pyrrole with carboxylated pyrrole (Py-COOH) in the presence of target protein CysC and multiwall carbon nanotubes (MWCNTs). After this, urea treatment was carried out to remove CysC from the molecularly imprinted polypyrrole matrix. The binding capability of CysC to the MIP was evaluated by DPV anodic peaks in the presence of ferro/ferricyanide. The DPV current decreased linearly when CysC concentration increased from 0.5 to 20.0 ng mL^−1^ and the LOD was found to be less than 0.5 ng mL^−1^. The sensor was also used to analyze CysC in spiked serum samples and yielded a promising response with recoveries of less than 3% variation. This technology has potential properties for on-the-spot detection of acute renal damage in terms of simplicity, cost-effectiveness, and sensitivity.

#### 5.1.4. Other electrochemical strategies

**Fig. 4:**
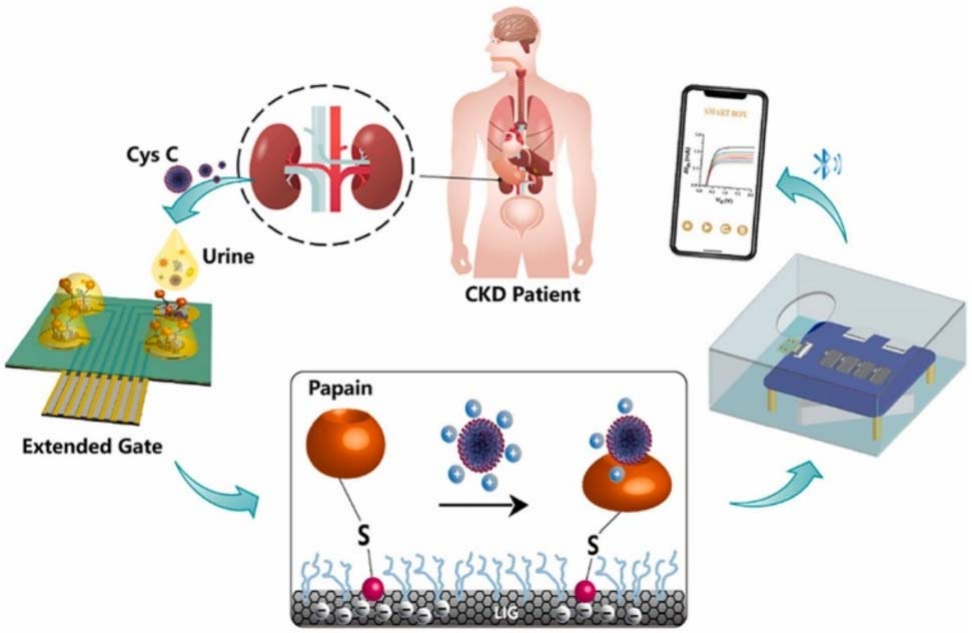
The schematics of the sensor working principle of FET based CysC sensor by using extended-gate field-effect transistor (EG-FET). Reproduced with permission from ref. 73. Copyright © 2024 Elsevier B.V.

Besides the conventional electrochemical sensing and capacitive sensing, various sensors have been developed for CysC detection such as field-effect transistor (FET), volt-ampere measurement and photo electrochemistry analysis. A study employing a silicon nanowire (SiNW)-based FET biosensor for the detection of CysC was conducted by Hu *et al.*^72^ A wafer-scale, highly controllable SiNW-FET with high sensitivity was made by using 13.5 nm SiNW through spacer image transfer (SIT) processes and channel doping optimization. The CysC antibodies were immobilized on the oxide layer of the SiNW by oxygen plasma treatment and silanization. When CysC binds with the antibody, the resistance of SiNW increases and was measured to detect the CysC concentration. The developed sensor showed a LOD of 0.25 ag mL^−1^ with a linear range of 1 ag mL^−1^ to 1 ng mL^−1^. The method stands out for its remarkable sensitivity, quickness, label-free nature and ability to identify early symptoms of kidney failure. Another FET-based CysC sensor was developed by Chen *et al.* by using extended-gate field-effect transistor (EG-FET).^73^ This sensor was comprised of a commercially available metal-oxide-semiconductor field-effect transistor (MOSFET) and a graphene electrode array in monolithic interface as shown in Fig. 4. The extended gate electrode coated with laser induced graphene loaded with AuNPs in the presence of adhesive material coating enhances the electrical performance of extended gate electrode. This was followed by modifying the surface with papain, which can combine with CysC to create specific protein complexes and thus causing decrease in the current value. This portable EG-FET sensor showed a promising linear range of 5 ag μL^−1^ to 50 ng μL^−1^ with low detection limit of 0.05 ag μL^−1^.

An innovative dual detection method for CysC in the blood plasma by surface-enhanced Raman spectroscopy (SERS) and electrochemistry was presented by Hassanain *et al.*^74^ Here, the gold-coated silicon nanopillar electrode was modified with thiol and CysC antibody to recognize the CysC specifically and was followed by buffer wash for the quick isolation of CysC. The isolated CysC was then subjected to rapid reduction of disulfide bonds, and the reduced protein could be easily immobilized on conductive gold coated silicon nanopillar substrate using strong gold–sulfur bonds. Following that, the electrochemical DPV and SERS measurements were used to quantify the reduced protein detection. A reproducible surface-enhanced Raman spectroscopy measurement is made possible by the oriented immobilization of CysC. The LOD of the developed sensor for SERS and DPV was 1 pM and 62.5 nM, respectively. These values satisfy the requirements for tracking renal failure and Alzheimer's disease.

In another work, the interdigitated electrodes were modified with carbon nanowire and papain and this was used as the detection probe for CysC.^75^ The immobilization of papain was done by covalent bonding between the carboxyl group of carbon nanowire and the amine group of papain. By measuring the voltage *vs.* current responses using a picoammeter, this papain-modified electrode surface was able to detect CysC concentration in the range of 100 pg mL^−1^ to 3.2 ng mL^−1^. The current response gradually increased with increase in the CysC concentration from 100 to 3200 pg mL^−1^ and the LOD was found to be 200 pg mL^−1^. The developed sensor was highly specific as other interfering molecules such as creatinine and gliadin are not properly binding to the immobilized papain.

In a different approach, an effective photo electrochemical immuno sensor was developed for the sensitive detection of CysC using titanium dioxide nanotube arrays (TNAs) after modifying with the CysC-specific nanobody.^76^ From a library of phage display nanobodies, they have isolated CysC-specific nanobodies. Under irradiation the TNAs produced by electrochemical anodization showed a large and stable photocurrent response. CysC was quantitatively detected in the range of 0.72 pM to 3.6 nM using the change in photocurrent that occurs when CysC binds to the CysC-specific nanobody attached to TNA. The created immunosensor demonstrated assay accuracy and a detection limit of 0.14 pM, indicating selectivity and stability. A comparison of all the electrochemically based sensors reported for CysC detection is presented in Table 1.

**Table 1:** A comparison of electrochemical sensors developed for the detection of CysC

Method	Electrode	Reaction	Signal	LOD	Detection range	Selectivity	Real sample	Ref.
Voltammetry-DPV	Sandwich assay by using a capture antibody modified SPCE	Alkaline phosphatase label in anti-CysC antibody induces a chemical reaction in addition of mixture of 3-IP and silver nitrate in the assay	Current increases	6.0 ng mL^−1^	10–100 ng mL^−1^	—	Serum	64
Voltammetry-DPV	Antibody modified graphene oxide-chitosan (GO-Chit) nanocomposite/GCE	Ferricyanide [Fe(CN)6]^3−^ mediated electron transfer	Current decreases	0.0078 mg L^−1^	1–10 mg L^−1^	Ascorbic acid, uric acid, citric acid, alanine, asparagine, tyrosine, nicotinamide adenine dinucleotide, cysteine, creatinine, and cystine (Cys)	Serum	65
Voltammetry-square wave voltammetry	Antibody modified polyethyleneimine-graphene oxide-ferrocene nanofilm/GCE	Ferrocene mediated the electron transfer studies	Current decreases	0.03 ng mL^−1^	0.1–1000 ng mL^−1^	—	Serum	66
Voltammetry-DPV	ZIF-8-Cu_1−*x*_Ni_*x*_(OH)_2_@Cu nanocomposite modified on PEDOT:PSS coated ITO electrode	CysC immobilization with antibodies	Current increases	33 pg mL^−1^	0.1–1000 ng mL^−1^	Ascorbic acid, creatinine, lipocalin 2, human serum albumin, hemoglobin	Serum	67
Voltammetry-cyclic voltammetry (CV), DPV	Papain immobilized on screen printed MWCNT electrode	Papain–CysC interaction	Current decreases	0.58 ng L^−1^	0.6 × 10^−5^–6.6 × 10^−3^ ng μL^−1^	Creatinine, albumin and gliadin	—	68
Voltammetry-DPV	AuNP/C60/GCE	Immunoreaction-driven DNA strand displacement	Current increases	3 fg mL^−1^	0.01 pg mL^−1^ to 30 ng mL^−1^	Influenza, thrombin, hemoglobin, and matrix metalloproteinase-2	Serum	69
Capacitance	Monoclonal antibodies modified polypyrrole/carbon nanotube nanohybrid film/Gold interdigitated electrode	Change in capacitance on CysC binding	Difference of the phase angle-increases	28 ng mL^−1^	0–300 ng mL^−1^	—	Serum	70
Voltammetry-CV, DPV	MIP based SPCE electropolymerizing pyrrole (Py) with carboxylatedPy (Py-COOH) in the presence of Cys-C and MWCNT and selective removal	Selective recognition of CysC with MIP	Current decreases	0.5 ng mL^−1^	0.5–20.0 ng mL^−1^	Creatine kinase-MB, ascorbic acid, creatinine and bovine serum albumin (BSA)	Serum	71
FET	CysC antibodies modified SiNW FET	CysC immobilization with antibodies	Current decreases	0.2529 ag mL^−1^	1 ag mL^−1^ to 1 ng mL^−1^	Recombinant human retinol binding protein	—	72
FET	BSA/Papain/Au NPs/adhesive/laser-induced graphene electrode	Papain–CysC interaction	Current decreases	0.05 ag μL^−1^	5 ag μL^−1^ to 50 ng μL^−1^	BSA, papain, uric acid, creatinine, urea, gliadin, glucose	Urine	73
Spectroelectrochemical-SERS, DPV	Gold nanopillar electrode	Thiol chemistry used to develop target-specific and recyclable extractor chip	Raman band intensity of reduced, DPV current increases	62.5 nM by DPV and 1 pM by SERS	0.0625 μm to 1 μm for DPV 100 nM to 1 pM for SERS	—	Plasma	74
Current-volt measurement	Carbon nanowire-modified surface with interdigitated electrode	Papain–CysC interaction	Current increases	200 pg mL^−1^	200 pg mL^−1^ to 3.2 ng mL^−1^	Creatinine and gliadin	—	75
Photoelectrochemical	CysC-specific Nb modified TiO_2_ nanotube arrays	CysC immobilization with nanobodies	Photocurrent decrease	0.14 pM	0.72 pM to 3.6 nM	Prostate-specific antigen, human chorionic gonadotropin, and α-fetoprotein	Serum	76

### 5.2. Optical methods for the detection of CysC

In optical methods for the detection of CysC, the biorecognition sensing element is combined with an optical transducer to form the optical response. The optical CysC sensors include fluorescence, colorimetric, luminescent biosensors, reflectometric interference spectroscopy-based sensor, as well as those sensors based on surface plasmon resonance. The following section discusses all these sensors in details.

#### 5.2.1. Colorimetric sensors

The inhibition activity of papain towards the CysC was used for the colorimetric detection of CysC by immobilizing the enzyme on multiwalled carbon nanotube (MWCNT).^77^ In this aspect, a diagnostic kit with casein beads and papain immobilized on MWCNT was developed. The inhibition of papain activity by CysC was used for the detection of CysC and the quantification was done by measuring the absorbance at 670 nm after adding the Folin–Ciocalteu reagent.

Lateral flow immunoassays (LFIA) have been explored as PoC devices as they allow fast, simple and cost-effective features along with high specificity, sensitivity, and low LOD. Zhang *et al.* reported quantitative LFIAs for the colorimetric detection of CysC by using two Au nanostructures: AuNPs and Au nanorods (AuNR).^78^ The conjugation of Au nanostructure-monoclonal antibodies was confirmed by UV-Vis spectroscopy and the authors have arrived at the optimum composition for the best LIFA performance by measuring the hydrodynamic radius of monoclonal antibodies-Au nanostructure conjugate through dynamic light scattering.

The LIFA signal intensity at various concentrations of CysC was monitored at optimum conditions and was used for the quantitative detection of CysC. The detection range was enhanced by the wash step to remove the unbound analyte to free up the space so that monoclonal antibodies–Au nanostructures conjugate could efficiently bind to the capture antibody-immobilized CysC. The authors found that AuNRs conjugates were more sensitive than AuNPs, despite AuNPs having greater test line signal intensities. The LOD of AuNRs based conventional and step-wash LFIA in dipstick format were found to be 0.08 mg L^−1^ and 0.35 mg L^−1^, respectively.

**Fig. 5:**
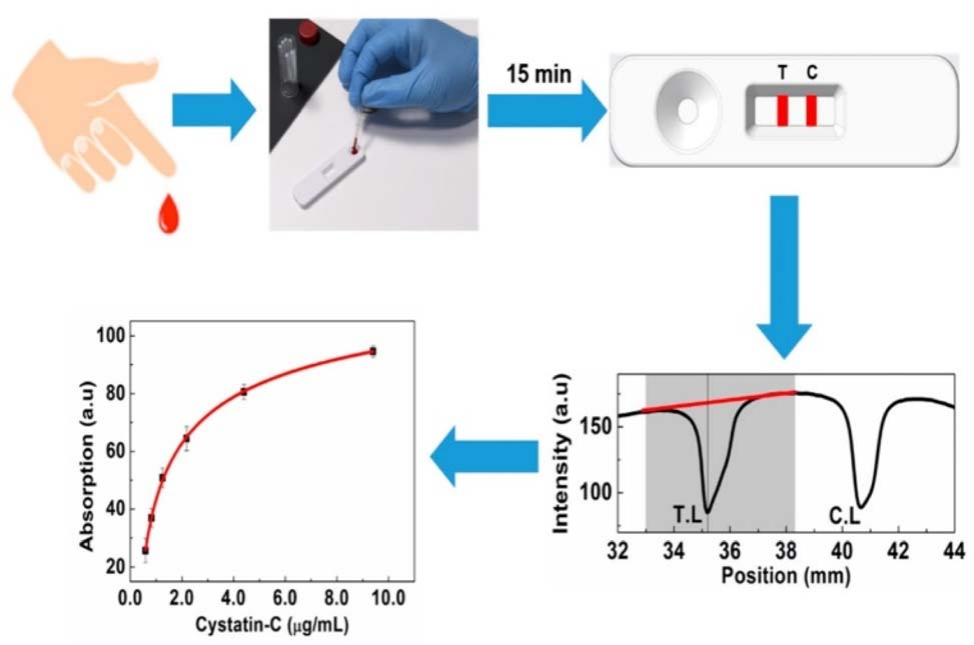
The schematic of the experimental procedure for the detection of CysC by using the developed LFIA strip. The red line represents the baseline correction and shaded area represents the tolerance. Reprinted with permission from ref. 79. Copyright © 2024 by the authors.

Recently, a colloidal gold-based LFIA device was developed for the quantitative detection of CysC with a minimum assay time of 15 min.^79^ The AuNP-CysC antibody conjugate was synthesized by covalent binding between carboxyl functionalized AuNP and EDC modified antibody by EDC-NHS chemistry. The binding between the CysC and antibody was measured colorimetrically. The schematic of the experimental procedure for the detection of CysC by using the developed LFIA strips is given in Fig. 5. The developed device is able to detect CysC in the range from 0.5 to 7.5 μg mL^−1^, with a LOD at 0.18 μg mL^−1^. The developed LFIA device exhibited satisfactory stability of more than 30 day when stored at 50 °C thus indicating the long shelf life at room temperature. In addition, the developed device did not require expensive readers or complex conjugation protocols, which helps in fast device development towards easy commercialization. Furthermore, the device has the potential to provide home-based diagnostics as it can be connected to a mobile phone.

**Fig. 6:**
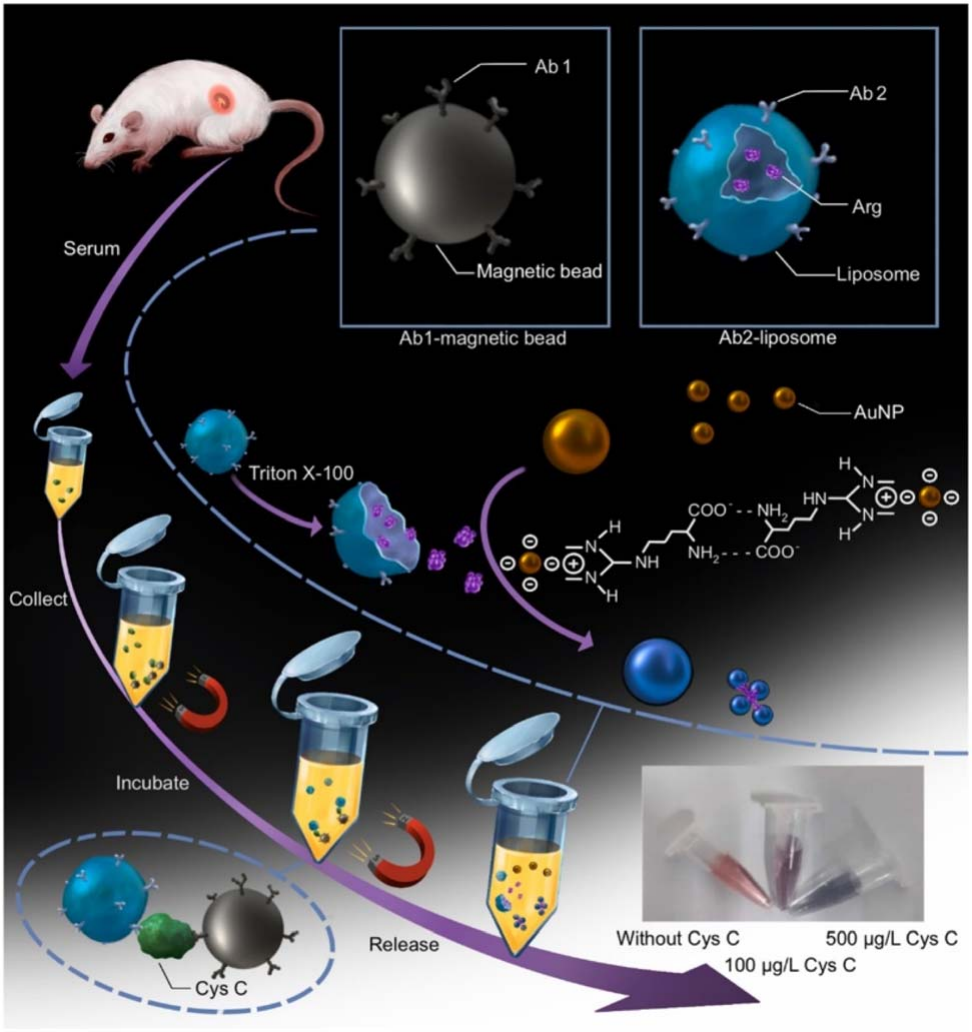
Schematic illustration of the principle and operation steps of CysC sensor made by a arginine liposome-amplified colorimetric immunoassay. Reprinted with permission from.^80^ Copyright © 2022 Elsevier B.V.

In another work, a arginine liposome based colorimetric assay was created for the detection of CysC.^80^ Here, arginine (Arg)-loaded liposomes and magnetic beads (MBs) were used as surfaces for the conjugation of CysC antibodies (Ab1 and Ab2), resulting in Ab1-MBs and Ab2-Arg-liposomes, accordingly. The schematic illustration of the principle and operation steps of CysC sensor made by a arginine liposome-amplified colorimetric immunoassay is given in Fig. 6. When CysC is present, it will be captured by Ab1-MBs and this was followed by the addition of Ab2-Arg-liposomes to form the Ab1-MBs-CysC-Ab2-Arg-liposomes immuno-sandwich complex. Triton X-100 was added as a last step to break the liposomes and release Arg, which could cause the aggregation of gold nanoparticles. Utilizing this discoloration, which has a linear range of 10–100 μg L^−1^, CysC can be detected quantitatively and visually.

#### 5.2.2. Fluorescence sensors

A fluorescence sensor for CysC was made by using glutaraldehyde functionalized nitrogen-doped graphene quantum dots (NGQDs) as a support material for the immobilization of papain.^81^ It was found that immobilization of papain on NGQDs has significantly enhanced the stability of papain as the activity immobilized papain is maintained by 80% for 150 min at 70 °C while that of free enzyme was only 55% after 50 min. The interaction between papain and CysC could be measured by fluorimetric studies and the sensor showed that it could able to detect CysC in the range between 0.05 ng L^−1^ to 50 ng L^−1^ with LOD of 0.05 ng L^−1^.

**Fig. 7:**
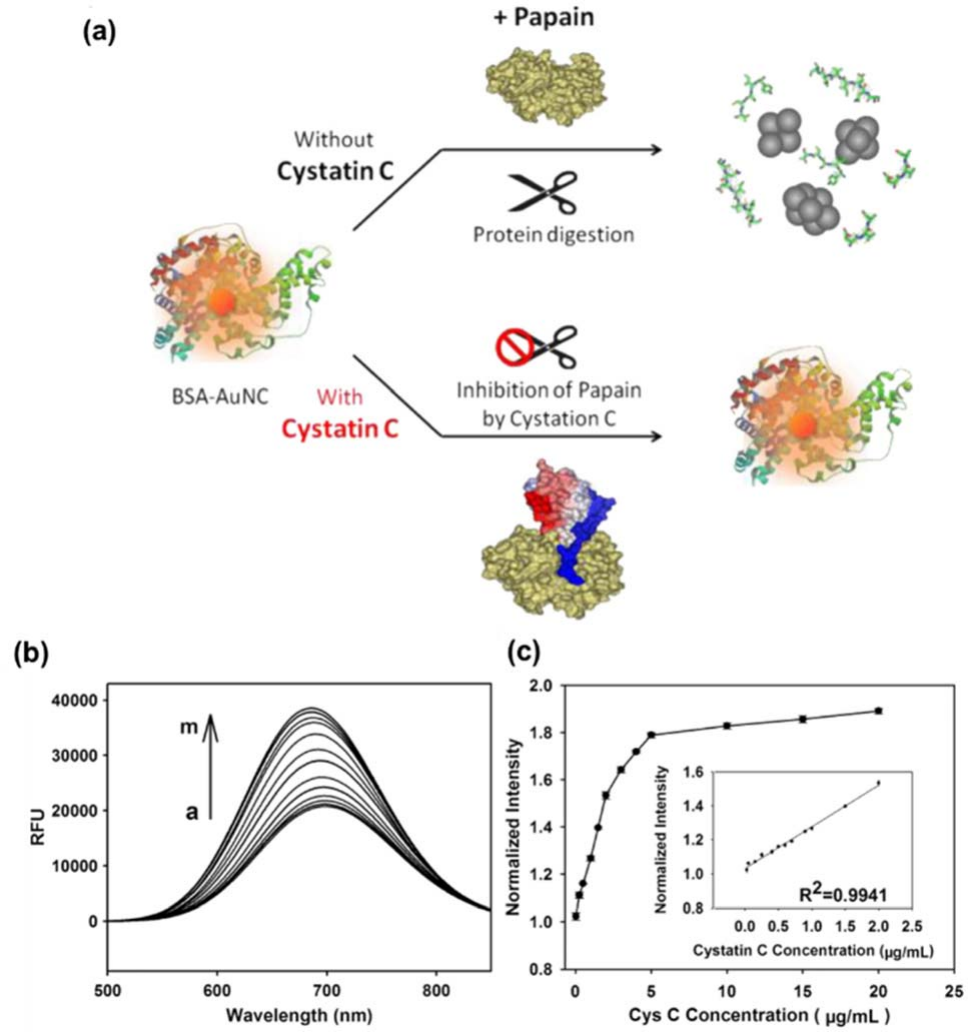
(a) Schematics of the BSA-AuNCs-based fluorescent sensor for the detection of Cys C (b) fluorescence responses of BSA-AuNCs in presence of papain and different concentrations of CysC. (c) Plot showing the relation between fluorescence intensity of BSA-AuNCs and concentration of CysC. Inset shows the calibration plot of the normalized intensity *versus* the concentrations of CysC in the range of 00.25 to 2.0 μg mL^−1^. Reprinted with permission from.^82^ Copyright © 2012 Elsevier B.V.

Lin *et al.* reported a simple, immune-independent and label-free fluorescent detection for CysC using BSA-stabilized Au nanoclusters (AuNCs).^82^ This method relied on the BSA scaffold degradation caused by the specific inhibition of papain activity by CysC and cysteine protease activity of papain. The fluorescence of BSA-AuNCs could be effectively quenched by papain, and restored by the presence of CysC.The concentration of CysC was determined by observing the turn-on fluorescence signal of the AuNCs–papain combination. The schematics showing the working principle of the sensor and the fluorescence responses of BSA-AuNCs with different concentrations of CysC as well as its calibration plot are given in Fig. 7. This novel protein-templated AuNCs based protease inhibitor assay was able to detect CysC concentrations in the range of 25 ng mL^−1^ to 2.0 μg mL^−1^ with high selectivity. The detection limit of the sensor was found to be 4.0 ng mL^−1^ and this was around 40 times better than commercial sensing approaches.

Tao *et al.* employed a similar method of hydrolyzing the BSA template with papain to create a label-free CysC sensor that used PbS@BSA quantum dots (QDs) that emit near-infrared (NIR) light.^83^ Pb^2+^ in PbS@BSA QDs was primarily bound to the amino and carboxyl groups of BSA, and the papain digestion process reduced the QDs' fluorescence. An analysis and optimization of the relationship between papain concentration and NIR fluorescent intensity was done. Before papain was combined with QDs, CysC could inhibit its protease activity while preserving the intensity of the fluorescence. This was used in the development of the CysC sensor, and it was discovered that the LOD was 0.02 μg mL^−1^. They have also found CysC in 50% serum, and they suggest that this is a good way to detect CysC quantitatively in comparison to other optical detection techniques, as well as for qualitative bioimaging.

In a similar approach of hydrolyzing the BSA, a novel fluorescence on-off sensor was made by combining using aggregation-induced emission (AIE) feature of 2-(4-bromophenyl)-3-(4-(4-(diphenylamino)styryl)phenyl) fumaronitrile (TPABDFN) and BSA.^84^ The BSA loaded with TPABDFN shows “On” state due to the restricted intramolecular motions. The BSA could be hydrolyzed by papain and this causes a decrease in the fluorescence. The presence of CysC prevents the template protein from being hydrolyzed by proteases, thus maintaining the fluorescence and the fluorescence intensity was utilized to successfully to quantify CysC. The developed sensor was able to detect CysC with a linear range of 12.5 ng mL^−1^ to 800 ng mL^−1^ and a LOD of 7.10 ng mL^−1^. In another work, an immune-pillars chips were used for fluorescent-based detection of CysC by using fluorophore labeled antibody on the micro beads.^85^ The micro beads assembled in the nanopillars provide the binding site for CysC and the fluorescence intensity was measured. The fluorescence intensity increased with increasing CysC concentration in the immuno-pillar chips and the sensor was able to detect CysC from 0 to 50 ng mL^−1^ with LOD of 3 ng mL^−1^. The developed immunochip showed a significant decrease in the reaction time (20 min) when compared to the current ELISA technique (240 min) and also needs only minimum quantity reagent for the sensing.

Fluorescence-based LFIA sensors have also been reported for the CysC detection towards PoC applications. An aptamer-antibody pair-based sandwich lateral flow immunoassay is proposed by Natarajan *et al.*^86^ They have fabricated the sensor by conjugating CysC selective aptamers to the organic dye Alexa fluor-647. In presence of CysC, a complex is formed between the aptamer and CysC, provided the LFIA strip's test zone is immobilized with CysC antibody. The developed assay was able to achieve the LOD of 0.013 μg μL^−1^, which is better than any antibody-based kit. In summary, the developed LFIA aptamers-based sensor provides a fast, sensitive, low cost POC sensor for CysC detection from human samples. A similar fluorescent-based LFIA sensor was developed in a sandwich format by using a fluorescent organic dye conjugated monoclonal antibody.^87^ By monitoring the fluorescence intensity with respect to the analyte content, the CysC level in the sample could be quantified. The calibration of the kit was linear in nature between 0.023–32 μg mL^−1^ and the LOD was found to be 0.023 μg mL^−1^.

#### 5.2.3. Fluorescence resonance energy transfer (FRET) based sensors

Non-radiative energy transfer from an excited donor fluorophore molecule to a nearby acceptor fluorophore molecule is termed as fluorescence-induced resonance energy transfer (FRET). Usually, the donor and acceptor molecules in a FRET-based biosensor are fluorescent proteins or fluorescent nanomaterials that are intended to be in close proximity to one another. A novel and straightforward FRET-based biosensor was effectively built to evaluate CysC with exceptional sensitivity by employing a DNase I-supported recycling amplification method.^88^ The biosensor utilizes graphene oxide (GO) as the efficient FRET receptor, which could bind with fluorophore-labelled aptamer. This binding protects aptamer from the hydrolytic action of DNase I. At this stage, the fluorescence is in the “OFF” state and will be changed to “ON” state when CysC is present due to the binding between CysC and aptamer. This will prevent aptamer from adhering to the surface and leave it vulnerable to digestion by DNase I. Following this, the fluorophore-labelled aptamer could be broken down by DNase I, allowing the CysC for use in the subsequent reaction. Thus, DNase I enzyme plays a crucial role in initiating the subsequent phase and generating signal amplification, which leads to high sensitivity of the sensor. A linear range of 0.625–20 ng mL^−1^ and a very low LOD of 0.16 ng mL^−1^ for CysC was obtained with this unique approach, which is almost three times lower than the detection limit obtained without DNase I.

#### 5.2.4. Surface plasmon resonance (SPR) based sensors

A microneedle that painlessly draws blood from a finger and detects CysC by using localized surface plasmon resonance (LSPR) biosensing technology was developed by Puttaswamy *et al.*^89^ They have developed lab-on-a-carbohydrate-microneedle with high aspect ratio and utilized LSPR paper-based substrates for the detection of CysC. The manufacturing of the microneedle is simple, adaptable, affordable, and readily scalable. The fluid channels in these microneedles are made entirely of maltose, which is then coated with polylactic-*co*-glycolic acid (PLGA). Because PLGA is porous, blood is drawn to the surface by capillary action and filtered out so that only the plasma in the blood reaches the biorecognition layer of the biodevice. The nitrocellulose paper is modified with AuNPs functionalized with CysC antibody by surface adsorption. The developed paper nanoplasmonic substrate could detect CysC through changes in the refractive index within 10 nm from the surface of the AuNPs. This was based on the assumption that the size of the antibody and the CysC complex would be less than 10 nm. Hence, the detection of CysC up to a concentration of 0.01 μg mL^−1^ could be achieved by the developed micro needle device.

Utilizing a specific interaction between CysC and immobilized papain in a solution, a SPR sensor was fabricated for the detection of CysC.^90^ An amino modified gold surface was used to immobilize papain by activating the surface with NHS and EDC. Amino modification of gold surface was achived by cysteamine so that it interacts better with the immobilized papain and makes it easier for CysC to bind. This sensor had a sensitive LOD of 0.09 μg mL^−1^ and a responsive range of 0 to 0.6 μg mL^−1^. A comparison examination using the PETIA method was carried out in order to validate these results and it showed good agreement. This biosensor could precisely measure the quantities of CysC in a range of biological fluids such as blood plasma, urine, and saliva. Another SPR-based sensor was developed by same research group by using bromelain or chymopapain or ficin for the specific CysC detection.^91^ The enzymes bromelain, ficin, and chymopapain are categorized as cysteine proteases, sharing a similar mechanism of action. In presence of CysC, the formation of an enzyme–inhibitor complex was formed by immobilized bromelain or chymopapain or ficin on the biosensor surface. As part of the optimization process, the effects of interaction pH and the influence of enzyme concentration on the SPR signal were examined. It is important to note that there was an outstanding degree of similarity between the calibration curves for the three different enzyme-based sensors. The developed sensor was able to detect CysC in the range of 0 to 0.6 μg mL^−1^ with a LOD of 0.1 μg mL^−1^. In addition, the developed sensor could accurately detect CysC levels in the blood plasma, urine, and saliva.

#### 5.2.5. Chemiluminescence based sensors

The design of the fast chemiluminescence sandwich immunoassay for CysC made use of mesoporous silica nanoparticles (MSN) loaded with dye and functionalized with signaling antibodies.^92^ Initially, they have modified the polystyrene plate reaction well with capture CysC antibody. A sandwich assay in the presence of CysC and functionalized MSN formed capture CysC antibody – CysC – functionalized MSN as shown in Fig. 5. After the sandwich immunoassay formation, acetone was added to get free dyes from the assay. Then the chemiluminescence assay was evaluated by introducing trichlorophenyl oxalate (TCPO)–H_2_O_2_–imidazole-fluorescent dye reaction system. The developed sandwich immunoassay was able to detect CysC in the range of 0.0035–0.5 ng mL^−1^ with LOD of 0.0029 ng mL^−1^. The results showed that this method is simple, rapid, ultra-sensitive and could detect CysC from multiple samples. This automated chemiluminescence analyzer could detect 96 wells continuously.

**Fig. 8:**
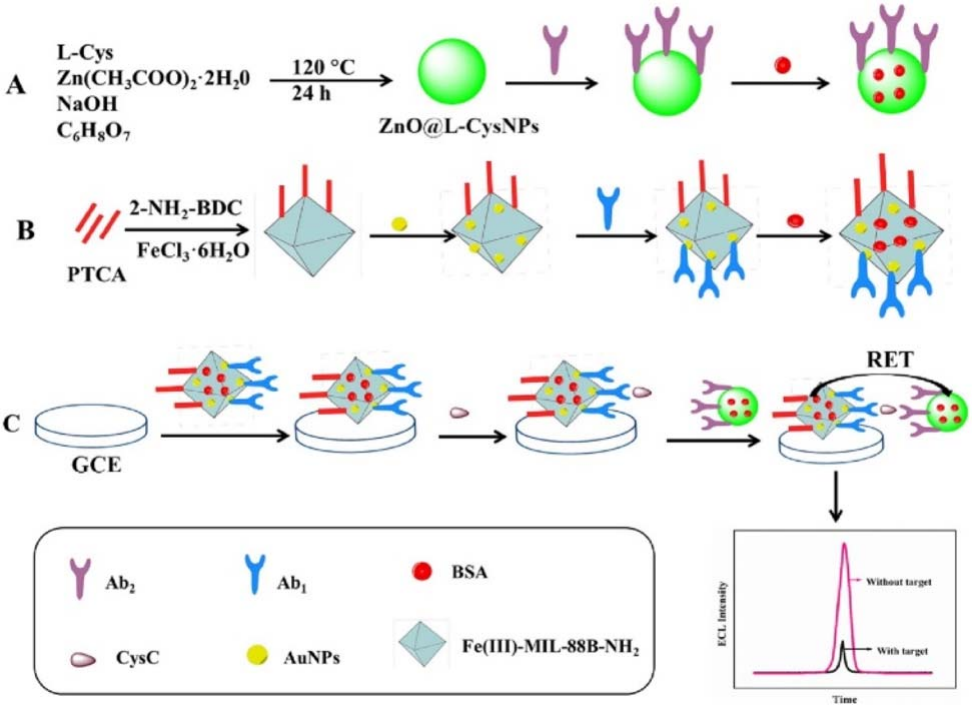
(A) Schematic showing the preparation of ZnO@L-CysNPs/Ab2 (A) and synthesis of PTCA@Fe(iii)-MIL-88B-NH_2_@Au/Ab1. (B) The preparation of ECL-FRET immunosensor as well as (C) the detection of CysC. Reprinted with permission from ref. 93. Copyright © 2023 Elsevier B.V.

Another chemiluminescence sensor for CysC detection was developed by using the perylenetetracarboxylic acid (PTCA) based donor and the ZnO@L-CysNPs as the acceptor.^93^ In order to create PTCA@Fe(iii)-MIL-88B-NH_2_@Au/Ab1, PTCA was first mixed with a Fe containing metal–organic framework (Fe(iii)-MIL-88B-NH_2_). This was followed by the *in situ* growth of AuNPs and antibody binding. This complex was drop casted on GCE so that CysC could bind to the Ab1. ZnO@L-CysNPs and Ab2 of CysC and BSA were combined in parallel to create the ZnO@L-CysNPs/Ab2 complex. Fig. 8 provides the schematics for the synthesis of PTCA@Fe(iii)-MIL-88B-NH_2_@Au/Ab1 and ZnO@L-CysNPs/Ab2, as well as the preparation of the ECL-FRET immunosensor. The specific interaction of the antigen with the antibody links the ZnO@L-CysNPs/Ab2 to the PTCA@Fe(iii)-MIL-88B-NH_2_@Au/Ab1 when CysC is present. Through a subsequent resonance energy transfer between the PTCA@Fe(iii)-MIL-88B-NH_2_@Au donor and the ZnO@L-CysNPs acceptor, this binding process may be able to quench the electrochemiluminescence (ECL) of PTCA. The developed ECL-resonance energy transfer (RET) based biosensor exhibited a linear range between 0.01 pg mL^−1^ and 100 ng mL^−1^ with a detection limit of 2.2 fg mL^−1^.

#### 5.2.6. Reflectometric interference spectroscopy based sensors

A label-free CysC biosensor has been developed by Bleher *et al.* by measuring the thickness of transducer surface by using the fundamentals of reflectometric interference spectroscopy (RIfS).^94^ In RIfS, the interference spectra of the sensitive layer changes as the optical thickness of the layer changes. In this work, the antigen–antibody interaction causes change in the interference spectra and this change is used to quantify CysC in human serum.

The sensor chip incorporates a glass transducer with a sensitive layer produced on its surface by immobilizing CysC, resulting in a stable platform for the detection. The second step involves incubating the sample with constant antibody concentrations and injecting the incubated sample onto the surface of the sensing platform. Using RifS, the antibody's binding to the immobilized CysC was identified. The amount of CysC in the sample has an inverse relationship with the binding response. The developed sensor was able to accurately quantified CysC in serum samples in the medically relevant range of 0.53–1.02 mg L^−1^. A comparison showing the characteristics of optical sensors developed for the detection of CysC is given in Table 2.

**Table 2:** A comparison showing the characteristics of optical sensors developed for the detection of CysC

Method	Sensing strategy/element	Reaction	Signal	LOD	Detection range	Selectivity	Real sample	Ref.
Absorbance	Papain modified MWCNT in solid support and casein beads	Protease assay and its inhibition by CysC	Increases		0.5–5 mg L^−1^	—		77
Colorimetric	Antibody conjugates of AuNPs and AuNRs	Lateral flow assay	Increases	AuNPs – 0.42 mg L^−1^ AuNRs – 0.35 mg L^−1^	Up to 12.5 mg L^−1^		Serum	78
Colorimetric	Antibody conjugates of AuNPs	Lateral flow assay	Increases	0.18 μg mL^−1^	0.5–7.5 μg mL^−1^	Hemoglobin, bilirubin, triglycerides, rheumatoid factor	Serum, blood	79
Colorimetric	Ab1-MBs and Ab2-Arg-liposomes	Arginine-liposome-amplified colorimetric immunoassay	Discoloration	4.32 μg L^−1^	10–100 μg L^−1^	BSA, FBS, Cr, blood urea nitrogen	Serum	80
Fluorescence	BSA protected AuNCs	Fluorescence quenching by papain, and restoration by the presence of CysC	Increases	4.0 ng mL^−1^	25 ng mL^−1^ to 2.0 μg mL^−1^	Thrombin, hemoglobin, glucose oxidase and trypsin	Urine	82
Fluorescence	PbS@BSA QDs	Fluorescence quenching by papain, and restoration by the presence of CysC	Increases	0.02 μg mL^−1^	0.05–2 μg mL^−1^	K^+^, Mg^2+^, Zn^2+^, Ca^2+^, Mn^2+^, uric acid, dopamine, glucose, fructose, lactose, lysine, cysteine, glutamic acid, tyrosine, tryptophan, alanine, urea, haemoglobin, DNA	Serum	83
Fluorescence	TPABDFN co loaded BSA	Aggregation-induced emission TPABDFN and BSA quenched by papain and restored by the coexistence of CysC	Increases	7.10 ng mL^−1^	12.5–800 ng mL^−1^	lipase, pancrelipase,neuraminidase, thrombin,IL-6,IL-10,TNF-α,and TGF-β	Urine	84
Fluorescence	Sandwich format by using a fluorescent organic dye conjugated monoclonal antibody	Sandwich lmmuno assay	Increases	3 ng mL^−1^	0–50 ng mL^−1^	—	—	85
Fluorescence	CysC selective aptamers conjugated to dye Alexafluor-647	Lateral flow assay	Increases	0.013 μg mL^−1^	0–120 μg mL^−1^	—	—	86
Fluorescence	Sandwich format by using a fluorescent organic dye conjugated monoclonal antibody	Lateral flow assay	Increases	0.023 μg mL^−1^	0.023–32 μg mL^−1^	BSA	Urine	87
Fluorescence resonance energy transfer	Graphene oxide-fluorophore-labelled aptamer and DNase I	Resonance energy transfer	Increases	0.16 ng mL^−1^	0.625–20 ng mL^−1^	BSA, kidney injury molecule-1, neutrophil gelatinase-associated lipocalin	Urine	88
Surface plasmon resonance	Nitrocellulose paper coated with AuNPs	CysC interaction with AuNPs	Red shifts in the LSPR wavelength	0.1 μg mL^−1^	0.1 to 3 μg mL^−1^	N-terminal pro B-type natriuretic peptide and BSA	Blood	89
Surface plasmon resonance	Papain modified gold surface	Papain–CysC interaction	Red shifts in the LSPR wavelength	0.09 μg mL^−1^	0–6 μg mL^−1^	—	Plasma, urine and saliva	90
Surface plasmon resonance	Bromelain or chymopapain or ficin modified gold surface	Bromelain or chymopapain or ficin–CysC interaction	Red shifts in the wavelength of the SPR	0.1 μg mL^−1^	0–0.6 μg mL^−1^	—	Plasma, urine and saliva	91
Chemiluminescence	Functionalized mesoporous silica nanoparticles	Sandwich immunoassay	Luminescence increases	0.0029 ng mL^−1^	0.0035–0.5 ng mL^−1^	—	Urine	92
Electrochemiluminescence resonance energy transfer	GCE modified with PTCA@Fe(iii)-MIL-88B-NH_2_@Au/Ab1	Resonance energy transfer	Luminescence decreases	2.2 fg mL^−1^	0.01 pg mL^−1^ to 100 ng mL^−1^	Prostate-specific antigen, carcinoembryonic antigen, bull serum albumin, carbohydrate antigen 125	Serum	93
Reflectometric interference spectroscopy	CysC coated glass plate	Binding inhibition assay with CysC antibodies	Optical thickness increases	—	0.53–1.02 mg L^−1^	—	Serum	94

### 5.3. Nuclear magnetic resonance based detection of CysC

Nuclear magnetic resonance (NMR) sensor application utilizes protons, which possess a magnetic moment and will orient along the direction of the field inside a strong magnetic field. The orientation of protons can be disturbed by a radio pulse with a specific resonance frequency. In presence of radio pulse, these excited spins will induce a radio frequency signal in which the amplitude of the signal is directly proportional to the number of spins in the sample. After this, the NMR signal of the spins relaxes exponentially and these signal relaxations (spin–lattice relaxation (T1) and spin–spin relaxation (T2)) could be used to characterize their physicochemical environment.

**Fig. 9:**
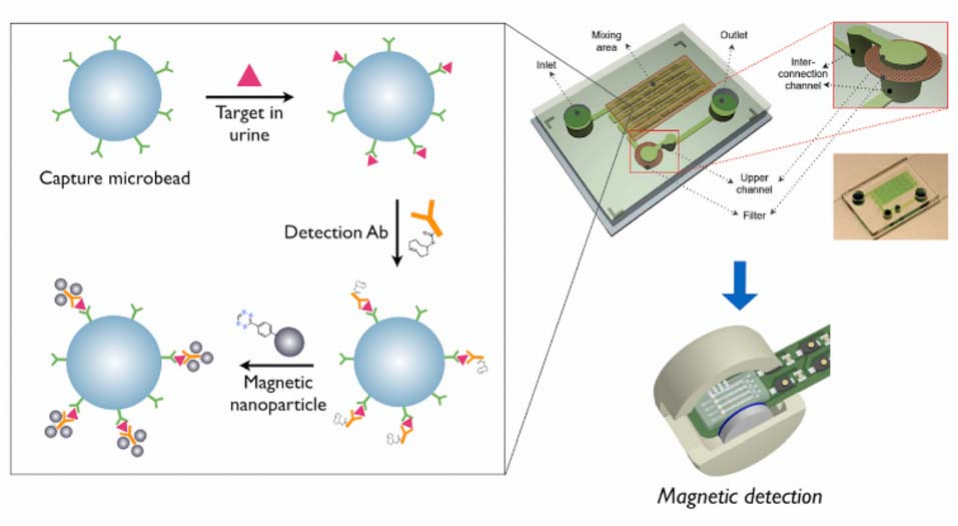
The schematics of the μUNPD assay for CysC detection. Firstly, capture antibody conjugated microbeads, followed by TCO conjugated detection antibodies, and finally amino modified magnetic nanoparticles, are added for the CysC detection from the urine sample on the microchip. Reprinted with permission from ref. 95. Copyright: © 2015 Chung *et al.*

Chung *et al.* developed a technique named micro-urine nanoparticle detection (μUNPD) approach to detect soluble protein markers KIM-1 and CysC in urine by using miniaturized nuclear magnetic resonance device.^95^ To begin with, polystyrene microbeads coated with capture antibodies are mixed with CysC containing sample. Following incubation, all unattached molecular components were removed by a washing method. After that, the detecting antibody conjugate after combing with *trans*-cyclooctene (TCO) was added to the mixture. After another round of washing, amino modified Fe_2_O_3_ magnetic nanoparticles are added for labeling. The amino modified Fe_2_O_3_ magnetic nanoparticles were bound to TCO-modified detection antibody conjugate. Then the tagged beads were tested for T2 relaxation using micro NMR device. The schematics of the μUNPD assay for CysC detection is given in Fig. 9. This method ensures that the CysC was recognized and measured properly in the urine sample. The method was used to detect CysC in the concentration rage of 300–10 000 ng mL^−1^ with a LOD of 20 ng mL^−1^. In another study, Wu *et al.* utilized non-contrast enhanced native T1 mapping of the renal cortex towards assessing renal fibrosis and found that a favorable correlation between T1 value and the concentration of CysC.^96^

## 6. Conclusion and future scope

This review focuses on the overview of the various CysC sensors including conventional and newly introduced techniques. To the best our best knowledge, this is the first review focused exclusively on CysC biosensors including conventional and newly introduced methods. Human CysC is a stable basic protein and is a critical cysteine protease inhibitor routinely generated by nearly every nucleated cell in the body. It was established that CysC is an accurate biomarker for GFR overcoming the challenges posed by measuring serum creatinine. Hence, the development of accurate and selective biosensors for the detection of CysC from human samples is extremely important for monitoring the GFR.

The conventional methods used for the CysC monitoring are PETIA, PENIA and ELISA while the newly introduced methods include mainly electrochemical, optical and NMR based techniques. The research on the development of biosensor for CysC detection is not fully explored as only few articles are published compared to other biosensors developed for other important biomarkers. The major bio recognition methods used in the development of these sensors are either antibody based, aptamer based or cysteine proteases based recognition with CysC. Among these, aptamer-based recognition methods are mainly used in conventional methods. Hence, there are ample opportunities for developing aptamer-based sensors considering their strong binding affinity with the target. The electrochemical and optical biosensors for CysC detection mainly use the binding between CysC and their specific antibodies or binding between CysC and cysteine proteases (CysC–papain binding).

The LOD of the developed sensors are promising as the normal range of blood CysC is 0.8–1.2 mg L^−1^. However, the performance should be maintained for commercialized PoC sensors for CysC. Additionally, the sensor response time should be as minimum as possible. Even though LFIA-based sensors are providing the sensor response within 15 min, it should be maintained for the real sample analysis. The sensors based on binding CysC with antibody or cysteine proteases have the potential to upgrade to device development and easy commercialization as it does not require complex conjugation protocols or expensive readers.

To overcome the challenges especially in producing deliverable and reproducible response without any interferences, there is a need to modify the sensor development strategy for optimizing the immobilization process of antibodies or cysteine proteases. This process will help to enhance the storage and operational stability of the sensor devices. Additionally, there is need to avoid the pre-treatment of biological samples for fast screening and ease-of-use. Furthermore, the sensor devices should have the potential to be connected with a mobile phone, which helps in providing home-based diagnostics. These developments are expected to revolutionize and commercialize the sensor devices for CysC diagnostics ideal for practical POC applications.

## Data availability

Data will be available as per request.

## Author contributions

JR and DG written the manuscript. JB and PAR done the review and editing, and supervision of the manuscript. All authors read and approved the final manuscript.

## Conflicts of interest

All authors declare no financial or non-financial competing interests.
